# Development and Functional Characterization of an Interesterified Fully Hydrogenated Rapeseed Oil/Sea Buckthorn Oil Fat System for Non-Tempered Confectionery Glazes

**DOI:** 10.3390/molecules31091407

**Published:** 2026-04-24

**Authors:** Askhat Dalabayev, Nazym Alzhaxina, Anar Kurmanbayeva, Mukhtar Tultabayev, Diana Temirova, Maussymzhan Amanzholova

**Affiliations:** Kazakh Scientific Research Institute of Processing and Food Industry LLP, Astana Z00X4F2, Kazakhstan; dalabaev_askhat@mail.ru (A.D.); anara121579@gmail.com (A.K.); yrath2510@gmail.com (M.T.); di.pandicorn6@gmail.com (D.T.); junesoul03@mail.ru (M.A.)

**Keywords:** interesterification, fully hydrogenated rapeseed oil, sea buckthorn oil, confectionery glaze, solid fat content, structured lipids

## Abstract

The development of alternative fat systems for confectionery glazes requires precise control of melting behavior, solid fat content, and rheological performance. In this study, binary fat systems based on fully hydrogenated rapeseed oil (FHRSO) and refined sea buckthorn oil (RSBO) were developed and modified by chemical interesterification for application in non-tempered confectionery glazes. Interesterified blends with FHRSO/RSBO ratios of 10/90, 20/80, and 30/70 were characterized in terms of fatty acid composition, trans fatty acid isomers, melting behavior, solid fat content (SFC), and rheological properties. The investigated systems were distinguished by a high content of palmitoleic acid (C16:1) derived from RSBO, while increasing FHRSO content led to higher saturated fatty acid levels, higher melting temperatures, and increased SFC values. Among the tested formulations, the FHRSO/RSBO 20/80 blend exhibited the most balanced functional profile, showing moderate melting characteristics, an SFC value of approximately 15% at 30 °C, and favorable Casson viscosity for glaze processing. A confectionery glaze prepared with this fat system showed good flow behavior during application, rapid setting at ambient temperature, and stable surface appearance during 30 days of storage. The results demonstrate that chemically interesterified FHRSO/RSBO systems, particularly the 20/80 formulation, represent a promising alternative lipid base for non-tempered confectionery glazes.

## 1. Introduction

Fat-based confectionery glazes are widely used to impart a glossy appearance, controlled melting behavior, pleasant snap, and a protective barrier against moisture and oxygen in confectionery products. Unlike tempered chocolate, the quality of fat-based glazes is governed by the formation of a structured fat network during cooling after processing. Consequently, glaze performance is determined by the interrelation between processing conditions (melting, mixing, cooling, and setting), thermal behavior of the lipid phase, and the resulting fat structure, which together control flow behavior during application, setting kinetics, final texture, and stability during storage [1,2,3].

In recent years, the design of tailored fat systems has become a central strategy in confectionery technology, driven by volatility in cocoa butter supply and increasing demand for alternative lipid materials with predictable functional properties [1,4]. Various vegetable fat blends and structured lipid systems have been investigated as cocoa butter substitutes or replacers, aiming to reproduce the desirable melting behavior and solid fat profile required for confectionery coatings and glazes [5,6].

A key technological requirement in modern confectionery fat formulation is the development of trans-free structured fats with adjustable melting profiles and controlled solid fat content. Among the available modification approaches, interesterification has been widely applied to redistribute fatty acids among triacylglycerol molecules without generating trans fatty acids, enabling the design of fats with targeted functional characteristics [7,8,9]. In particular, systems combining fully hydrogenated oils with liquid vegetable oils have been shown to benefit from interesterification, as the process reduces excessive hardness of the hardstock while improving plasticity, melting behavior, and processability [10,11,12].

From a processing-oriented perspective, interesterification modifies the triacylglycerol distribution, leading to smoother melting transitions and more homogeneous thermal behavior compared to non-interesterified blends [13,14]. Differential scanning calorimetry (DSC) is commonly employed to characterize melting transitions and thermal properties of structured lipid systems and to support formulation decisions in fat-based product development [15,16]. These thermal characteristics are particularly critical for confectionery glazes, where temperature-dependent solidification behavior determines viscosity during coating or enrobing, setting rate after application, and mechanical integrity of the final glaze layer.

Alongside advances in structured vegetable fats, increasing attention has been directed toward the use of non-traditional plant oils with distinctive chemical compositions. Sea buckthorn (*Hippophae rhamnoides*) oil is known for its unusual fatty acid composition, particularly its high content of palmitoleic acid (C16:1), which is rarely present in significant quantities in conventional vegetable oils [17,18,19]. In addition, sea buckthorn oil contains bioactive minor components such as carotenoids and tocopherols, which may contribute to oxidative stability and nutritional value [20].

Previous studies have demonstrated that refined sea buckthorn oil retains characteristic compositional features while offering improved stability and compatibility with food lipid systems [21,22]. Despite these promising characteristics, the application of sea buckthorn oil in structured confectionery fats remains limited.

In the present study, we investigate a binary interesterified fat system composed of fully hydrogenated rapeseed oil (FHRSO) and refined sea buckthorn oil (RSBO) designed for application in confectionery glazes. The aim of this work was to establish relationships between processing conditions, lipid composition, thermal behavior, and functional properties of the resulting fat systems. Particular attention was given to the evaluation of fatty acid composition, trans fatty acid isomers, melting characteristics, solid fat content, rheological properties, and glaze performance during storage [23,24,25,26,27].

## 2. Results and Discussion

### 2.1. Fatty Acid Composition and Trans Fatty Acid Isomers

The experimental fatty acid composition of the starting oils fully hydrogenated rapeseed oil, refined sea buckthorn oil and their interesterified blends is summarized in Table 1.

As observed, the overall fatty acid profile of the blends was strictly governed by the relative proportions of the starting oils.

Refined sea buckthorn oil (RSBO) was characterized by a unique and dominant presence of palmitoleic acid (C16:1, 43.8%), which is a rare component for most vegetable fats but acts as a signature for *Hippophae rhamnoides*. In contrast, the fully hydrogenated rapeseed oil (FHRSO) hardstock consisted almost entirely of stearic acid (C18:0, 89.8%), providing the necessary high-melting saturated backbone [7,8].

A progressive increase in FHRSO content from 10 to 30 wt.% resulted in a significant rise in total saturated fatty acids (Σ SFA) from 35.4% to 48.5%, primarily due to the contribution of stearic acid. This was accompanied by a corresponding decrease in monounsaturated (Σ MUFA) and polyunsaturated (Σ PUFA) fractions. Despite the dilution, palmitoleic acid remained a major component even in the 30/70 blend (32.1%), ensuring the retention of the fluidity and reduced viscosity characteristic of sea buckthorn lipids [17,18,19].

Statistical analysis confirmed that chemical interesterification did not alter the qualitative or quantitative fatty acid profile compared to the initial physical mixtures. This proves that the significant changes observed in the thermal and rheological behavior of the 10/90, 20/80, and 30/70 samples (discussed in the following sections) are attributable to the redistribution of acyl groups within the triacylglycerol (TAG) structure rather than changes in the fatty acid building blocks themselves. Furthermore, the trace levels of trans-isomers detected by FTIR-ATR confirm that the processing conditions did not induce cis–trans isomerization, supporting the nutritional safety of the developed fat systems.

The content of trans fatty acid isomers in the interesterified FHRSO/RSBO fat systems remained low and did not show a systematic increase with increasing FHRSO content (Table 2).

**Figure 1:**
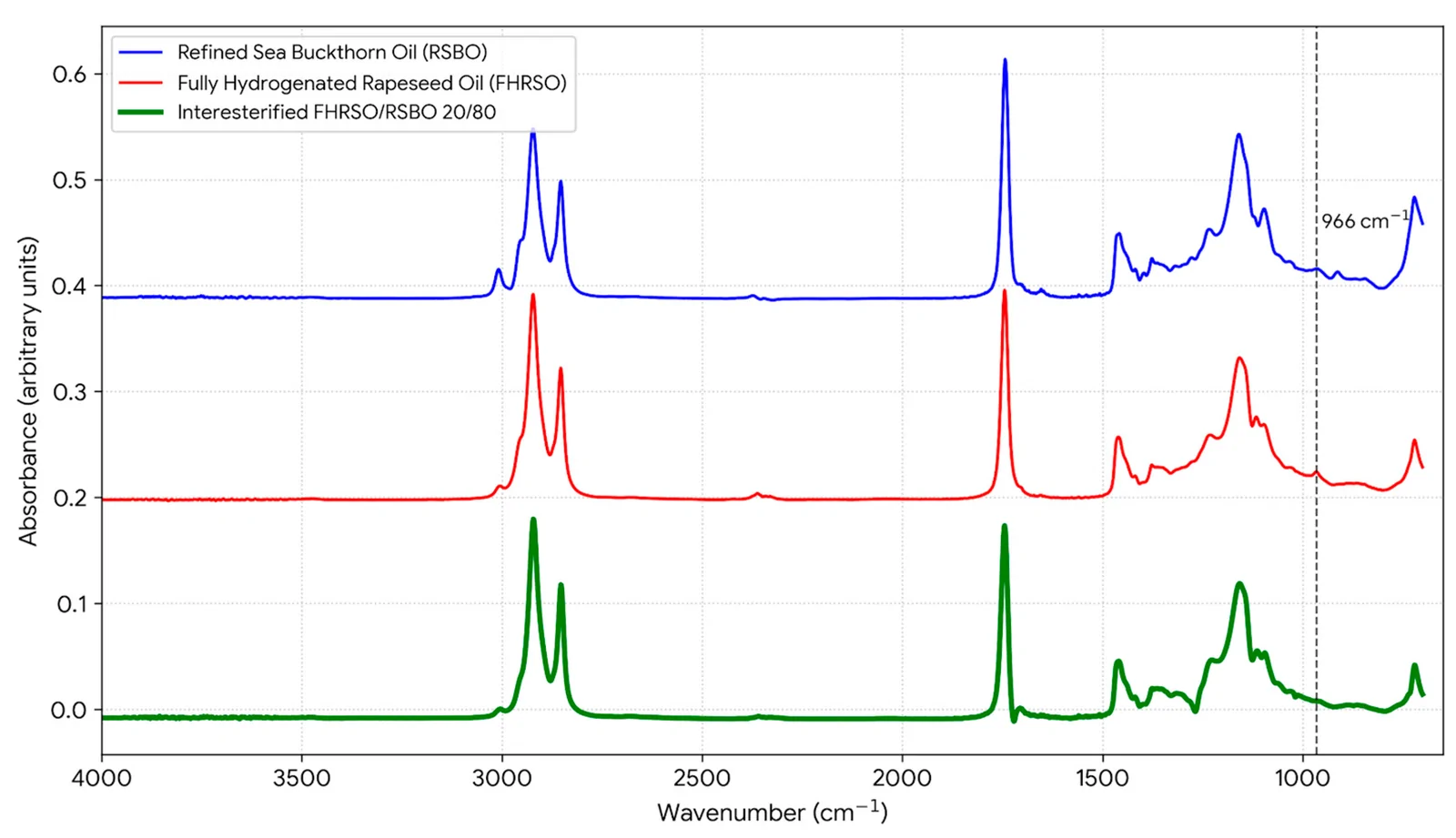
FTIR-ATR spectra of refined sea buckthorn oil (RSBO, blue), fully hydrogenated rapeseed oil (FHRSO, red), and chemically interesterified FHRSO/RSBO 20/80 blend (green).

Figure 1 shows the FTIR-ATR spectra of RSBO, FHRSO, and the chemically interesterified FHRSO/RSBO 20/80 blend. The absence of new absorption bands and no significant change in the intensity of the band at 966 cm^−1^ after interesterification confirm that the chemical reaction did not induce cis–trans isomerization or the formation of new chemical bonds, consistent with the expected rearrangement mechanism.

The content of trans fatty acid isomers was also evaluated in the interesterified fat systems. The results showed that trans fatty acids remained at very low levels (<2%), confirming that chemical interesterification does not significantly generate trans isomers when fully hydrogenated oils are used as hardstocks [23,24].

The observed values were within the range typically reported for natural vegetable oils when determined by FTIR-based methods. Importantly, chemical interesterification did not lead to the formation of additional trans isomers, confirming that the applied modification strategy preserves the cis configuration of unsaturated fatty acids.

### 2.2. Thermal Behavior of Interesterified FHRSO/RSBO Fat Systems

The melting behavior of the interesterified FHRSO/RSBO fat systems was investigated by differential scanning calorimetry to evaluate the influence of hardstock content on thermal properties relevant to confectionery glaze processing. Characteristic melting parameters, including onset temperature (T_onset), peak melting temperature (T_peak), and melting enthalpy (ΔH), are summarized in Table 3.

All DSC measurements were performed in at least triplicate, and the reported values are expressed as mean ± standard deviation. The observed shifts in T_onset and T_peak (approximately 4 °C per 10 wt.% increase in FHRSO content) are consistent with the progressive increase in saturated triacylglycerol content (Table 1) and reflect the structuring effect of the fully hydrogenated rapeseed oil. Although the differences between neighboring blends are moderate, they are statistically significant and functionally meaningful for tailoring the melting behavior of non-tempered confectionery glazes.

The thermal properties of the interesterified fat systems (Table 3) showed significant differences (*p* < 0.05) depending on the FHRSO/RSBO ratio. As the concentration of the hard fat fraction (FHRSO) increased, a consistent and statistically significant shift in melting temperatures and enthalpy was observed.

The blend with a 10/90 ratio exhibited the lowest onset melting temperature (27.2 ± 0.1 *^a^*). This relatively low value suggests a high liquid oil fraction at room temperature, which would likely result in a soft, oily texture and poor structural stability of the final glaze. Conversely, the 30/70 ratio showed a peak melting temperature (42.4 ± 0.2 *^c^*) that significantly exceeds human body temperature. Such high-melting profiles are generally undesirable in confectionery applications as they lead to incomplete melting in the mouth and a persistent ‘waxy’ sensation.

The 20/80 ratio was identified as the optimal composition. It provides a balanced melting profile with a peak temperature of 38.2 ± 0.1 °C, ensuring sufficient hardness and stability during storage while maintaining acceptable sensory properties and melting behavior close to physiological conditions. This choice is further supported by the solid fat content (SFC) data and rheological analysis, which confirm the suitability of the 20/80 blend for high-quality non-tempered glazes.

In parallel with the increase in melting temperatures, the melting enthalpy (ΔH) increased with increasing FHRSO content. This behavior is consistent with previously reported relationships between saturated triacylglycerol content and the thermal properties of structured lipid systems, where increasing the proportion of high-melting TAGs leads to higher melting enthalpy and melting temperatures [28]. The gradual increase in ΔH confirms that chemical interesterification effectively redistributes fatty acids among triacylglycerol molecules while preserving the ability to tune the overall solid fat structure of the fat systems through compositional adjustment.

Despite the increase in melting temperatures, the interesterified FHRSO/RSBO systems containing up to 30 wt.% FHRSO exhibited melting peak temperatures below 45 °C. This feature is particularly important for confectionery glaze applications, as it ensures complete melting at typical processing temperatures (45–50 °C) while allowing rapid solidification upon cooling to ambient conditions. The absence of excessively high-temperature melting transitions within this composition range indicates that the systems remain processable and do not exhibit excessive rigidity.

Preliminary DSC thermograms of blends with higher FHRSO contents (40–50 wt.%) exhibited a pronounced high-temperature melting peak (>55 °C), associated with excessive hardness and poor processability at typical glaze application temperatures (45–50 °C). Therefore, the composition range was deliberately limited to ≤30 wt.% FHRSO to maintain practical applicability.

Experimental DSC thermograms obtained for higher FHRSO contents (e.g., 50 wt.% FHRSO) revealed a dominant high-temperature melting peak above 55 °C, associated with excessive hardness and poor processability. These observations confirm the exclusion of FHRSO levels above 30 wt.% from further formulation development and support the selected composition window for confectionery glaze applications.

Overall, the DSC results demonstrate that chemical interesterification enables controlled modification of melting behavior in FHRSO/RSBO fat systems by balancing the rigid structural contribution of the fully hydrogenated hardstock with the plasticizing effect of refined sea buckthorn oil. The observed thermal trends provide a basis for interpreting the solid fat content and viscosity behavior of the systems, which are discussed in the following sections.

### 2.3. Solid Fat Content of Interesterified FHRSO/RSBO Fat Systems

**Figure 2:**
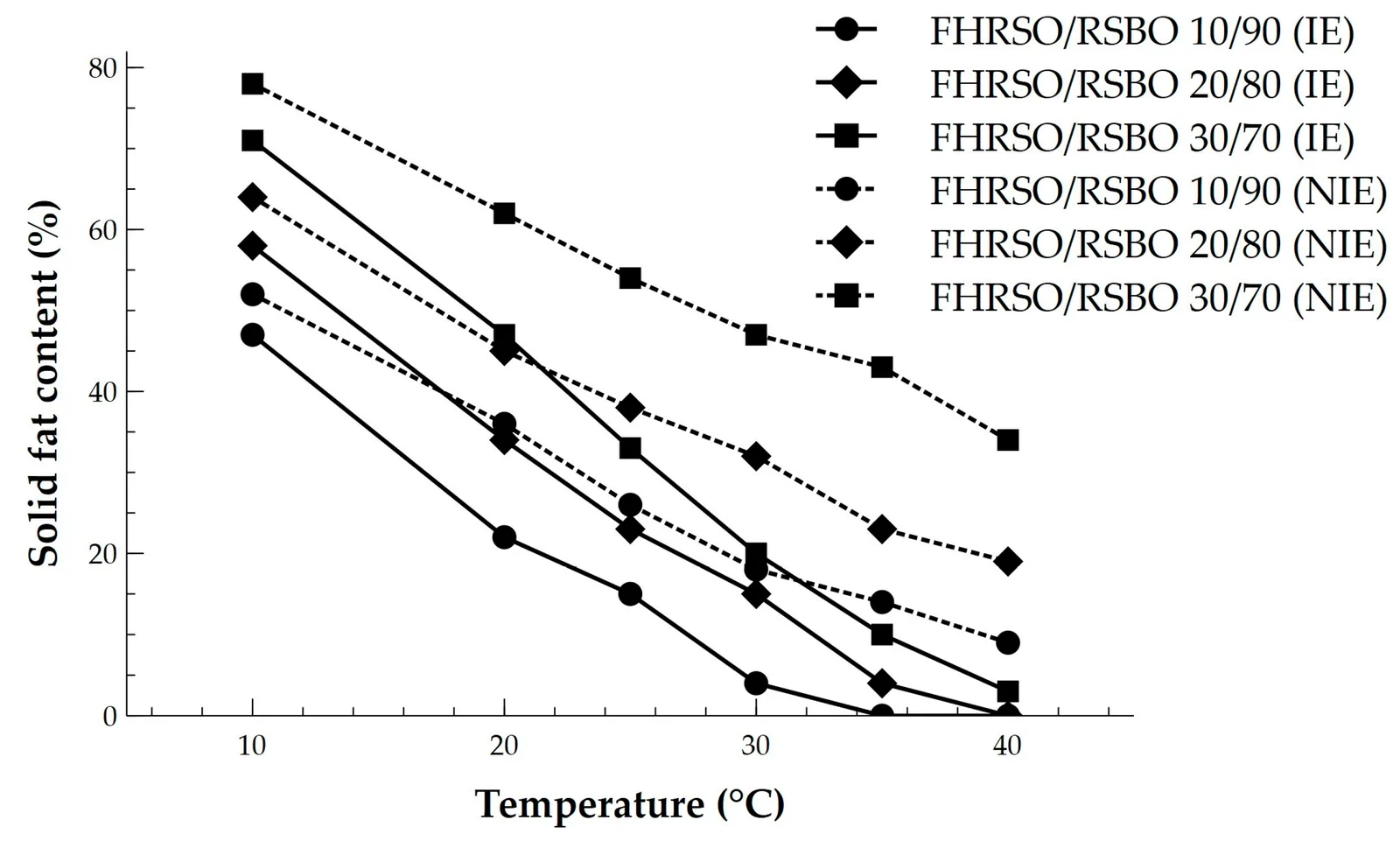
Solid fat content (SFC) profiles of corresponding non-interesterified (NIE) and interesterified (IE) FHRSO/RSBO fat systems at different temperatures.

To substantiate the benefits of chemical modification, the solid fat content (SFC) profiles of interesterified blends (IE) were compared with their corresponding non-interesterified (NIE) physical blends (Figure 2).

The NIE physical blends exhibited significantly higher SFC values across the entire temperature range (10–40 °C). For instance, at 30 °C, the non-interesterified physical blend 20/80 sample maintained an SFC of approximately 36%, whereas the interesterified IE 20/80 sample showed a reduction to 15%.

This downward shift in the SFC curve is a direct result of the redistribution of fatty acids within the triacylglycerol (TAG) molecules. Chemical interesterification breaks down the high-melting trisaturated TAG clusters (mainly triastearin from FHRSO) and incorporates unsaturated fatty acids from the sea buckthorn oil into the structure. This molecular rearrangement leads to a more gradual melting profile and improved plasticity. The lower SFC at 35–40 °C for the IE blends compared to the NIE counterparts is crucial for ensuring a smooth, non-waxy mouthfeel, while the stable solid phase at 20 °C provides the necessary structural integrity for the glaze.

At intermediate temperatures (20–25 °C), corresponding to glaze setting and storage conditions, SFC values ranged from 22 to 47% at 20 °C and from 15 to 33% at 25 °C. These values indicate sufficient structural strength of the fat matrix to maintain glaze integrity while avoiding excessive hardness.

Particularly important for confectionery glaze applications is the SFC at approximately 30 °C, which strongly influences the balance between structural stability and melt-in-the-mouth characteristics [29]. In the present study, the SFC at 30 °C increased from 4% in the FHRSO/RSBO 10/90 (IE) blend to 15% and 20% for the FHRSO/RSBO 20/80 (IE) and 30/70 (IE) systems, respectively. The latter two compositions fall within the typical range considered optimal for fat-based confectionery coatings, providing sufficient structural rigidity while maintaining acceptable processing properties.

At higher temperatures (35–40 °C), SFC values decreased sharply for all compositions, indicating progressive melting of the solid fat phase. Complete melting was observed for the 10/90 and 20/80 systems at 40 °C, while the 30/70 blend retained only a small residual solid fraction (3%). Such behavior is advantageous for glaze processing, ensuring complete melting at typical application temperatures and facilitating smooth flow during coating.

Overall, the SFC profiles demonstrate that adjusting FHRSO content within the 10–30 wt.% range enables effective tuning of the temperature-dependent solid fat fraction. The resulting balance between structural stability and melting behavior is consistent with the DSC results and provides a structural basis for the rheological properties of the molten glaze systems discussed in the following section.

Based on the obtained SFC profiles, the FHRSO/RSBO 20/80 (IE) system exhibited the most balanced structural characteristics for confectionery glaze applications. Similar SFC profiles have been reported for structured vegetable fat systems designed for confectionery coatings, where approximately 10–20% solid fat at 30 °C is considered optimal for achieving a balance between structural stability and good melting behavior [30,31]. At 30 °C, this composition showed an SFC value of approximately 15%, which falls within the range typically considered optimal for fat-based coatings, providing sufficient structural stability while maintaining good meltability and processing behavior. In contrast, the FHRSO/RSBO 10/90 (IE) system exhibited insufficient solid fat at this temperature, whereas the FHRSO/RSBO 30/70 (IE) blend showed comparatively higher rigidity across the investigated temperature range.

Considering these results, the FHRSO/RSBO 20/80 (IE) composition was identified as the most promising formulation. Therefore, subsequent investigations in this study were focused on the fat system containing 20 wt.% FHRSO and 80 wt.% RSBO.

The SFC profile strongly influences the rheological behavior of fat systems, since the amount of dispersed solid fat particles controls viscosity and flow properties.

### 2.4. Rheological Properties of the Confectionery Glaze Based on the FHRSO/RSBO 20/80 Fat System

The rheological behavior of the glaze was adequately described by the Casson model, and the calculated yield stress and plastic viscosity values are presented in Table 4.

**Figure 3:**
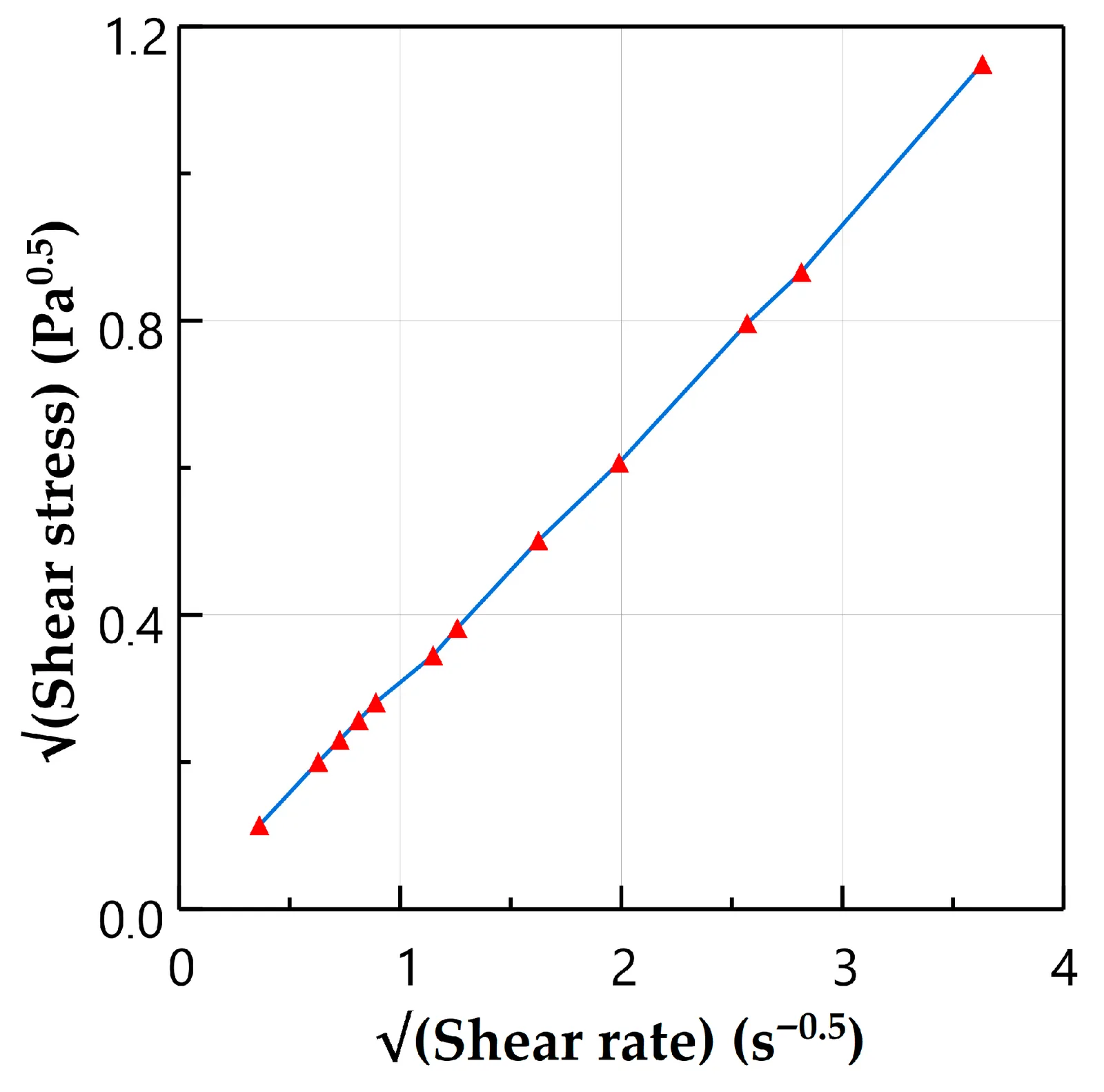
Rheological flow curves of the confectionery glaze based on the interesterified FHRSO/RSBO 20/80 fat system.

The calculated Casson plastic viscosity of the glaze was 0.097 Pa·s, indicating favorable flow behavior under processing conditions. At the same time, the Casson yield stress was close to zero, suggesting that the glaze did not exhibit pronounced resistance to the initiation of flow. Such rheological behavior is advantageous for coating applications, as it facilitates spreading and uniform distribution of the molten glaze over the product surface. The relationship between shear stress, apparent viscosity, and shear rate is presented in Figure 3.

The rheological parameters of the interesterified fat systems were determined using the Casson model, which is the standard approach for evaluating the flow properties and coating performance of confectionery glazes [32]. These values (yield stress and plastic viscosity) are crucial for ensuring the stability and uniform thickness of the glaze layer during application.

The observed viscosity characteristics are consistent with the previously established thermal and structural properties of the selected fat system. In particular, the FHRSO/RSBO 20/80 composition exhibited an SFC of 15% at 30 °C, which provided sufficient structural contribution without causing excessive rigidity. In addition, DSC analysis showed that the system melted completely within the temperature range relevant to glaze preparation. The combination of moderate solid fat content and controlled melting behavior contributed to the formation of a glaze with good processability and stable flow characteristics.

The influence of the lipid phase composition on the rheological behavior can be explained by the high correlation between fatty acid profiles and viscosity. According to Yalcin et al. (2012) [33], there is a strong positive correlation (R = 0.89) between the content of monounsaturated fatty acids (MUFA) and the viscosity of vegetable oils. In our study, the substantial proportion of palmitoleic acid (C16:1), which is a key MUFA in sea buckthorn oil, played a significant role in defining the flow resistance of the fat system. This relationship confirms that the unique fatty acid profile of RSBO directly contributes to the reduced apparent viscosity of the interesterified blends compared to traditional hard fats [33]. At the same time, the presence of the fully hydrogenated rapeseed oil fraction ensured sufficient structuring to maintain glaze setting and stability after application.

Overall, the rheological results confirm that the interesterified FHRSO/RSBO 20/80 fat system provides the most suitable balance between structural integrity and flowability among the investigated compositions. This supports the selection of the 20/80 formulation as the optimal lipid basis for confectionery glaze production.

### 2.5. Performance and Storage Stability of the Confectionery Glaze

The technological performance of the confectionery glaze formulated with the interesterified FHRSO/RSBO 20/80 fat system was evaluated under conditions relevant to industrial glaze application. The molten glaze exhibited stable flow behavior during mixing and application, forming a uniform coating layer on the model substrate.

At the processing temperature of 45–50 °C, the glaze demonstrated good spreadability and uniform coverage, which is consistent with the rheological properties determined in the previous section. The relatively low Casson plastic viscosity and the absence of significant yield stress facilitated smooth flow and ensured homogeneous distribution of the glaze during application.

After application, the glaze solidified within approximately 5–10 min at ambient temperature (18–20 °C), forming a smooth and glossy surface with uniform texture. The rapid setting behavior can be attributed to the balanced solid fat content profile of the FHRSO/RSBO 20/80 fat system, which provided sufficient solid fat content at room temperature while maintaining adequate meltability during processing.

Storage stability of the glaze-coated samples was evaluated under static conditions at 18–20 °C for 30 days. Throughout the storage period, the glaze maintained its structural integrity, surface gloss, and uniform appearance, with no visible surface defects or textural heterogeneity observed. The absence of visible deterioration indicates satisfactory stability of the lipid matrix during storage.

According to the expert assessment, the glaze based on the FHRSO/RSBO 20/80 blend maintained high glossiness (score 4.8 ± 0.2) throughout the 30-day storage period at 20 °C. No significant surface defects or heterogeneity were observed. This high stability is attributed to the optimized solid fat content (SFC) and the formation of a stable crystal network during the interesterification process, which prevents the migration of liquid oils to the surface and subsequent loss of gloss.

The observed technological performance confirms that the selected interesterified FHRSO/RSBO 20/80 fat system provides a suitable balance between melting behavior, structural stability, and rheological properties required for confectionery glaze production. The combined results of thermal analysis, solid fat content profiling, and rheological characterization demonstrate that the developed lipid system can serve as an effective alternative fat base for non-tempered confectionery coatings.

## 3. Materials and Methods

### 3.1. Materials

Fully hydrogenated rapeseed oil (FHRSO) was supplied by Maslo-Del LLP (Almaty, Kazakhstan). Sea buckthorn fruits (*Hippophae rhamnoides* L.) were purchased from a local market (Astana, Kazakhstan). The fruits were dried, ground, and subjected to hexane extraction at 60 °C for 5 h using a Soxhlet-type apparatus. The resulting crude sea buckthorn pulp oil was subsequently refined to remove undesirable impurities using a laboratory-scale neutralizing and bleaching reactor (Scientific Research Instrument, Vadodara, India).

Analytical standards, including a fatty acid methyl ester mix (Supelco 37 component FAME Mix), trielaidin, and triolein, as well as sodium methoxide (catalyst for interesterification) and n-hexane, were purchased from Sigma-Aldrich (St. Louis, MO, USA). All other chemicals and solvents used in the experiments were of analytical grade and used without further purification. Fully hydrogenated rapeseed oil was used as a structure-forming hardstock, while refined sea buckthorn oil served as the liquid oil component. Interesterification was applied to modify the triacylglycerol distribution and tailor the thermal and functional properties of the binary fat system for confectionery glaze applications.

### 3.2. Chemical Interesterification Procedure

Chemical interesterification of the binary FHRSO/RSBO fat system was performed to modify the triacylglycerol distribution and tailor the thermal and functional properties of the lipid matrix. A total of 200 g of the fat blend was placed in a 500 mL borosilicate glass laboratory reactor equipped with an overhead mechanical stirrer. Prior to the reaction, the fat mixture was dried under reduced pressure at 130 °C and a residual pressure of 4 kPa for 30 min to remove moisture and volatile impurities.

After drying, the fat blend was cooled to 80 °C, and chemical interesterification was initiated by the addition of 2.4 g of powdered sodium methoxide as a catalyst. The reaction was carried out at 110 °C under continuous stirring at 500 rpm for 60 min. Upon completion of the reaction, interesterification was terminated by the addition of 10 mL of distilled water preheated to 60 °C, ensuring deactivation of the catalyst.

The resulting reaction mixture was subsequently filtered under vacuum using filter paper to remove solid residues. The filtered fat was then dried under the same conditions as those applied prior to interesterification to remove residual moisture. The obtained chemically interesterified FHRSO/RSBO fat systems were stored under controlled conditions and used for further physicochemical characterization and preparation of model fat-based confectionery glazes.

### 3.3. Design and Composition of Interesterified FHRSO/RSBO Fat Blends

The design of the lipid system was aimed at developing a technologically suitable fat composition for confectionery glazes by combining a rigid structure-forming hardstock with a functional liquid oil component. Fully hydrogenated rapeseed oil (FHRSO) was selected as the hardstock due to its high content of saturated triacylglycerols and high melting characteristics, which provide structural strength and thermal resistance [7,10].

However, FHRSO is inherently very rigid, and excessive incorporation of this hardstock into fat systems can lead to increased hardness and elevated melting temperatures, which are undesirable for glaze processing and sensory perception [11,12].

Refined sea buckthorn oil (RSBO) was incorporated as the major component of the binary system to modulate rigidity and ensure adequate flow behavior at processing temperatures. RSBO is distinguished from many conventional vegetable oils by a relatively high content of palmitic acid (C16:0) and palmitoleic acid (C16:1), which contribute to both plasticity and partial solidification behavior of lipid systems [17,18,19].

Based on these considerations, interesterified FHRSO/RSBO fat systems were prepared at FHRSO/RSBO mass ratios of 10/90, 20/80, and 30/70. This composition range was selected to provide sufficient structural contribution from the hardstock while avoiding excessive rigidity associated with FHRSO contents above approximately 40 wt.% [8,11]. These ratios were selected after preliminary DSC and SFC screening to ensure the resulting fat systems remained suitable for glaze processing and application.

Chemical interesterification was applied to redistribute fatty acids among triacylglycerol molecules and improve compatibility between FHRSO and RSBO, resulting in more homogeneous melting behavior and improved functional performance compared with simple physical blends [9,13]. The resulting interesterified FHRSO/RSBO fat systems were thus designed to balance rigidity, melting behavior, and flow characteristics in accordance with the requirements of fat-based confectionery glaze production. This rational design provides the basis for subsequent evaluation of fatty acid composition, trans isomer content, thermal behavior, solid fat content, viscosity, and glaze performance, which are discussed in the following sections.

### 3.4. Preparation of Confectionery Glazes

Fat-based confectionery glazes were prepared using the chemically interesterified FHRSO/RSBO fat system as the lipid phase. The glaze formulation consisted of 31.2 wt.% interesterified fat blend, 16.2 wt.% cocoa powder, 51.0 wt.% powdered sugar, 1.5 wt.% lecithin, and 0.1 wt.% vanillin.

Prior to glaze preparation, the interesterified fat blend was melted at 45–50 °C to obtain a homogeneous liquid phase. Half of the total amount of lecithin (0.75 wt.%) was added to the molten fat and mixed thoroughly. Subsequently, the dry ingredients (cocoa powder, powdered sugar, and vanillin) were gradually incorporated into the fat phase under continuous mechanical stirring. The total mixing time was 20 min to ensure uniform dispersion of solid particles.

Five minutes before the end of mixing, the remaining portion of lecithin (0.75 wt.%) was added to adjust flow behavior and improve dispersion stability. Particle size reduction was performed in a single grinding step to achieve an average particle size in the range of 20–40 μm. The prepared glaze was used in molten form and exhibited setting within 5–10 min when cooled at 18–20 °C.

The resulting confectionery glaze was characterized by a glossy surface, uniform texture and high stability during storage, making it suitable for subsequent evaluation of rheological behavior, functional performance, and storage-related quality changes.

### 3.5. Analytical Methods

#### 3.5.1. Fatty Acid Composition

The fatty acid composition of the interesterified FHRSO/RSBO fat systems was determined by gas chromatography after conversion of fatty acids into their corresponding methyl esters (FAME). Fatty acid methyl esters were prepared according to the method proposed by Rodrigues et al. [34], with minor modifications where applicable.

FAME analysis was performed using a gas chromatograph (Agilent 7890B, Agilent Technologies, Santa Clara, CA, USA) equipped with a flame ionization detector (FID) and a highly polar capillary column CP-Sil 88 (100 m × 0.25 mm i.d., 0.20 μm film thickness). Helium was used as the carrier gas. The injector temperature was set to 250 °C, and the FID temperature was maintained at 200 °C. A sample volume of 1 μL was injected under the specified operating conditions.

Individual fatty acids were identified by comparing their retention times with those of a standard fatty acid mixture analyzed under identical chromatographic conditions. The fatty acid composition was expressed as the relative percentage of each fatty acid with respect to the total identified fatty acids. The analysis was conducted to verify that chemical interesterification did not alter the overall fatty acid profile of the FHRSO/RSBO blends and that observed differences in thermal and functional properties were associated with redistribution of fatty acids among triacylglycerol molecules rather than changes in fatty acid composition.

#### 3.5.2. Determination of Trans Fatty Acid Isomers

The content of trans fatty acid isomers in oils and fats was determined according to the AOCS Official Method Cd 14d-99 using Fourier transform infrared spectroscopy with attenuated total reflectance (FTIR-ATR). Infrared absorption spectra of calibration standards and fat samples were recorded using an IRSpirit-TX spectrometer (Shimadzu, Kyoto, Japan) equipped with a Quest ATR Accessory GS10800 Series (Specac, Orpington, UK) featuring a zinc selenide (ZnSe) ATR crystal.

Fat samples were placed directly onto the ATR crystal, which was maintained at 65 °C to ensure complete coverage and homogeneous contact between the sample and the crystal surface. Each sample was measured in triplicate. Spectra were collected in the range of 700–4000 cm^−1^ with 64 scans at a spectral resolution of 4 cm^−1^. Air was used as the background reference. The ATR crystal was thoroughly cleaned after each measurement.

The integrated absorbance area in the spectral region between 990 and 945 cm^−1^, corresponding to trans double-bond vibrations, was calculated using LabSolutions IR Version 2.33 software (Shimadzu, Kyoto, Japan). Quantification of trans fatty acid isomers was performed by comparison with calibration standards using linear regression. The analysis was conducted to verify that chemical interesterification did not result in the formation of trans fatty acid isomers in the FHRSO/RSBO fat systems.

#### 3.5.3. Thermal Analysis (DSC)

The thermal properties of the interesterified FHRSO/RSBO fat systems and the corresponding reference samples were analyzed using a differential scanning calorimeter DSC 1 (Mettler Toledo, Greifensee, Switzerland). The instrument was calibrated for temperature and enthalpy using indium as a standard prior to measurements.

Approximately 8–10 mg of each fat sample was completely melted and homogenized to eliminate previous thermal history and then placed into a hermetically sealed aluminum pan. An empty sealed aluminum pan was used as the reference. All DSC measurements were carried out under a nitrogen atmosphere at a flow rate of 50 mL min^−1^.

Heating scans were performed from −20 °C to 70 °C at a constant rate of 5 °C min^−1^. Melting thermograms were recorded, and characteristic thermal parameters, including onset temperature, peak temperature, and melting enthalpy (ΔH), were determined using the DSC 1/500 STARe evaluation instrument software. Each analysis was performed at least in triplicate. Characteristic temperatures (onset and peak) were determined using the instrument software with a precision of 0.1 °C

The melting behavior derived from DSC analysis was used to evaluate the effect of chemical interesterification and blend composition on the thermal characteristics of the fat systems and to assess their relevance for processing and storage conditions of fat-based confectionery glazes.

#### 3.5.4. Solid Fat Content

The solid fat content (SFC) of the interesterified FHRSO/RSBO fat systems and reference samples was determined using a nuclear magnetic resonance (NMR) spectrometer Minispec mq one (Bruker Corporation, Bremen, Germany). Measurements were performed using the direct method in accordance with the AOCS Official Method Cd 16b-93.

SFC values were recorded sequentially at temperatures of 10, 20, 25, 30, 35, and 40 °C. Prior to measurement, samples were conditioned according to the standard protocol to ensure thermal equilibrium at each temperature. The obtained SFC profiles were used to characterize the temperature-dependent solidification behavior of the fat systems and to assess their suitability for processing, setting, and storage of fat-based confectionery glazes.

#### 3.5.5. Viscosity Measurements (Casson Model)

The flow properties of the molten confectionery glazes were evaluated using viscosity measurements performed with a rotational viscometer Brookfield DV2T (AMETEK Brookfield, Middleboro, MA, USA). Measurements were carried out at controlled temperatures corresponding to glaze application conditions. Prior to analysis, glaze samples were equilibrated at the measurement temperature to ensure steady-state flow conditions.

Apparent viscosity values were recorded over a range of shear rates and processed using the Casson model, which is widely applied for chocolate and fat-based confectionery systems. Shear stress–shear rate data were used to construct Casson plots, and Casson viscosity values were calculated accordingly. Data processing and calculations were performed using experimental viscosity data obtained from the viscometer.

Each measurement was performed at least in triplicate. In the present study, Casson viscosity was used as a key technological parameter to assess the influence of interesterified FHRSO/RSBO fat systems on flow behavior and processability of the confectionery glazes.

#### 3.5.6. Evaluation of Glaze Performance and Storage Stability

The performance of the confectionery glazes was evaluated after application and setting under controlled conditions. Molten glazes were applied to model substrates and allowed to solidify at 18–20 °C. The setting behavior was assessed based on the time required for complete solidification, while surface appearance, gloss, and uniformity of the glaze layer were visually evaluated. These parameters were used as practical indicators of appropriate flow behavior during application and effective structure formation during cooling.

The visual performance and stability of the confectionery glazes were evaluated by a trained sensory panel consisting of 5 experts from the laboratory staff. The evaluation was conducted in a sensory room under standardized D65 daylight lighting. To ensure objectivity, a blind testing method was used, where samples were labeled with three-digit random codes. The following parameters were assessed:

Glossiness (Loss of gloss): Defined as the intensity of light reflection from the glaze surface. A transition from a bright, reflective surface to a dull or matte appearance was recorded as a loss of gloss.

Surface defects: Identified as the presence of visible cracks, fat bloom (white or greyish spots), or air bubbles.

Heterogeneity: Defined as the unevenness of the glaze layer or the presence of visible solid aggregates/crystals on the surface. Each parameter was evaluated on a 5-point scale, where 5 represented excellent quality (no defects) and 1 represented poor quality (unacceptable defects).

Storage stability of the confectionery glazes was evaluated under static storage conditions at 18–20 °C for a period of 30 days. During storage, the glazed samples were periodically inspected for changes in surface appearance, including loss of gloss, development of surface defects, or visual heterogeneity of the glaze layer. The absence of visible surface deterioration was considered indicative of satisfactory storage stability.

The selected evaluation criteria were intended to reflect the technological performance of fat-based confectionery glazes under realistic storage conditions. The observed performance during application, setting, and storage was interpreted in relation to the thermal behavior, solid fat content, and viscosity characteristics of the interesterified FHRSO/RSBO fat systems determined in the present study.

### 3.6. Statistical Analysis

All analytical measurements were performed at least in triplicate unless stated otherwise. Experimental data were expressed as mean values ± standard deviation. Statistical analysis was carried out using standard spreadsheet and statistical software, STATGRAPHICS Centurion 19 (Statgraphics Technologies, Inc., The Plains, VA, USA).

Differences between samples were evaluated using one-way analysis of variance (ANOVA). Statistical significance was assessed at a confidence level of 95% (*p* < 0.05). Where applicable, statistical analysis was applied to support interpretation of differences in thermal behavior, solid fat content, viscosity, and fatty acid composition among the interesterified FHRSO/RSBO fat systems.

Qualitative performance characteristics of the confectionery glazes, including surface appearance, setting behavior, and storage stability, were evaluated descriptively and interpreted in relation to the quantitative analytical results.

## 4. Conclusions

This study demonstrated the feasibility of developing functional fat systems for non-tempered confectionery glazes based on binary blends of fully hydrogenated rapeseed oil and refined sea buckthorn oil modified by chemical interesterification. The investigated systems were characterized by a distinctive fatty acid profile associated with sea buckthorn oil, particularly the high content of palmitoleic acid (C16:1), which contributed to improved fluidity while maintaining an adequate structural contribution to the lipid matrix.

An increase in FHRSO content from 10 to 30 wt.% led to higher melting temperatures, increased melting enthalpy, and higher solid fat content, confirming the structuring role of the fully hydrogenated hardstock. Among the investigated formulations, the FHRSO/RSBO 20/80 system exhibited the most balanced functional characteristics, combining moderate melting behavior, an SFC value of approximately 15% at 30 °C, and favorable rheological properties described by the Casson model.

The confectionery glaze prepared with the selected FHRSO/RSBO 20/80 fat system showed good processability, uniform coating behavior, rapid setting at ambient temperature, and stable appearance during storage. These findings indicate that the developed interesterified FHRSO/RSBO fat system is a promising alternative lipid base for confectionery glaze applications and may provide a practical approach for designing structured fat systems from regionally available vegetable oils. Further studies focused on detailed triacylglycerol composition and phase-transition behavior would provide additional insight into the structure–function relationships of the developed fat systems.

## Figures and Tables

**Table 1:** Fatty acid composition (wt.%) of starting oils (FHRSO, RSBO) and their interesterified blends.

Fatty Acid	FHRSO	RSBO	FHRSO/RSBO 10/90	FHRSO/RSBO 20/80	FHRSO/RSBO 30/70
C14:0 (Myristic)	4.4	0.3	0.7	1.1	1.5
C16:0 (Palmitic)	ND ^1^	26.1	23.5	20.9	18.3
C16:1 (Palmitoleic)	ND ^1^	43.8	41.2	36.6	32.1
C18:0 (Stearic)	89.8	2.5	11.2	20.0	28.7
C18:1 cis (Oleic)	3.6	11.8	10.1	9.4	8.6
C18:2 cis (Linoleic)	0.1	13.5	12.2	10.8	9.5
C18:3 cis (Linolenic)	0.1	0.6	0.6	0.5	0.5
Σ SFA	95.5	28.9	35.4	42.0	48.5
Σ MUFA	3.6	55.6	51.3	46.0	40.7
Σ PUFA	0.2	14.1	12.7	11.3	9.9

^1^ ND stands for not detected.

**Table 2:** Content of trans fatty acid isomers in interesterified FHRSO/RSBO fat blends.

Sample	Trans Fatty Acid Isomers (%)
FHRSO/RSBO 10/90	1.1 ± 0.2
FHRSO/RSBO 20/80	1.5 ± 0.1
FHRSO/RSBO 30/70	0.9 ± 0.1

Values are expressed as mean ± standard deviation (*n* = 3).

**Table 3:** DSC melting parameters of interesterified FHRSO/RSBO fat systems.

FHRSO/RSBO	T_onset (°C)	T_peak (°C)	ΔH (J/g)
10/90	27.2 ± 0.1 ^a^	34.1 ± 0.2 ^a^	32.4 ± 0.5 ^a^
20/80	31.4 ± 0.2 ^b^	38.2 ± 0.1 ^b^	42.1 ± 0.4 ^b^
30/70	35.1 ± 0.1 ^c^	42.4 ± 0.2 ^c^	54.8 ± 0.6 ^c^

Values are expressed as mean ± standard deviation (*n* = 3). Superscript letters (^a, b, c^) within the same column indicate significant differences (*p* < 0.05) according to Tukey’s HSD test.

**Table 4:** Rheological parameters of the confectionery glaze based on interesterified FHRSO/RSBO 20/80 fat system calculated by the Casson model.

Sample	Casson Yield Stress τ_0_ (Pa)	Casson Plastic Viscosity ηc (Pa·s)	R^2^
FHRSO/RSBO 20/80 glaze	0.013	0.097	0.99

## Data Availability

The original results presented in the study are included in the article; any further inquiries can be directed to the corresponding author.
